# Patterns of physical activity in Obstructive Sleep Apnoea and their association with sleepiness

**DOI:** 10.1007/s11325-025-03314-2

**Published:** 2025-04-04

**Authors:** By Jack Callum, Yu Sun Bin, Kate Sutherland, Amanda Piper, Kristina Kairaitis, John Wheatley, Philip de Chazal, Brendon J. Yee, Emmanuel Stamatakis, Peter A. Cistulli, on behalf of the Sydney Sleep Biobank Investigators

**Affiliations:** 1 Department of Respiratory and Sleep Medicine, Royal North Shore Hospital, Reserve Rd, St Leonards, NSW 2065 Australia; 2 Charles Perkins Centre, Faculty of Medicine and Health, University of Sydney, Sydney, Australia; 3 Department of Respiratory Medicine, Royal Prince Alfred Hospital, Camperdown, Australia; 4 Ludwig Engel Centre for Respiratory Research, The Westmead Institute for Medical Research, Westmead, Australia; 5 Department of Respiratory and Sleep Medicine, Westmead Hospital, Westmead, Australia; 6 Charles Perkins Centre, Faculty of Engineering, University of Sydney, Sydney, Australia; 7 Woolcock Institute of Medical Research, Macquarie University, Sydney, Australia; 8 Mackenzie Wearables Research Hub, Charles Perkins Centre, University of Sydney, Sydney, Australia; 9 School of Health Sciences, Faculty of Medicine and Health, University of Sydney, Sydney, Australia

**Keywords:** Obstructive Sleep Apnoea, Exercise, Wakefulness, Sleepiness

## Abstract

**Objectives:**

Excessive daytime sleepiness (EDS) is a key symptom of Obstructive Sleep Apnoea (OSA). Both EDS and OSA affect, and are affected by, physical activity (PA). This study explores physical activity patterns in OSA patients and the association between activity and EDS.

**Methods:**

This is a retrospective analysis of cross-sectional data from the Sydney Sleep Biobank, which recruited sleep clinic patients between August 2018 and June 2022. Participants aged > 18 years with untreated OSA were included, while those with other sleep disorders or whose medication/work affected sleepiness were excluded. PA was assessed with the International Physical Activity Questionnaire (IPAQ), with intensity quantified by metabolic equivalent of task (MET). Subjective daytime sleepiness with quantified by the Epworth Sleepiness Scale (ESS).

**Results:**

Of 487 patients with OSA, 21% reported low (< 600 MET-min/week), 32% medium (600–2999 MET min/week), and 47% high PA ( > = 3000 MET-min/week). Participants with mild OSA were the most likely to be in the high PA group. ESS was not significantly associated with physical activity nor OSA severity, after adjustment for sex, age, body mass index, and sleep duration. Consideration of a potential interaction between physical activity and OSA severity did not change these results. However, in subgroup analysis of women only, severe OSA and medium and high levels of PA were linked to higher ESS scores.

**Conclusions:**

Greater physical activity was associated with higher daytime sleepiness in women, but not men. However, further research is needed to reproduce these findings using objective measures of physical activity and to examine if physical activity has direct benefits for daytime symptoms of OSA beyond sleepiness.

**Supplementary Information:**

The online version contains supplementary material available at 10.1007/s11325-025-03314-2.

## Background

Excessive daytime sleepiness represents one of the most recognisable and clinically significant feature of Obstructive Sleep Apnoea (OSA), with excessive sleepiness being a strongly recommended indication for treatment listed by the American Academy of Sleep Medicine [1]. OSA is a disease affecting approximately one billion individuals worldwide [2]. OSA is characterized by recurrent episodes of partial or complete upper airway obstruction during sleep, leading to intermittent hypoxemia and sleep fragmentation, which result in excessive daytime sleepiness—defined as ‘daily episodes of an irrepressible need to sleep or daytime lapses into sleep [3]. The effect of this excessive daytime sleepiness extends beyond individual patients to public health, economic productivity and societal influences [2].

Physical activity (PA) includes a range of movement behaviours of varying intensities. Exercise captures only dedicated periods of high exertion whereas physical activity incorporates moderate activity (such as gentle cycling) as well as time spent walking. There is evidence to show that acute and chronic exercise positively affect sleep architecture, enhancing slow-wave sleep and modulating rapid eye movement (REM) [4, 5]. The timing of this activity may be important as exercise within 4 h of sleep can evoke a stress response and compromise sleep quality, though this has been an inconsistent finding [6–8]. While exercise and physical activity positively impacts mood and sleep quality, its influence on excessive daytime sleepiness remains relatively unexplored [9, 10]. Moreover, a bidirectional relationship is seen whereby excessive daytime sleepiness curtails physical activity due to diminished motivation.

Despite the pivotal role of PA in health, there is little research on physical activity patterns in OSA. Existing evidence suggests a potential association between increased physical activity with reduced daytime sleepiness and anxiety, alongside enhanced sleep quality in individuals with OSA [11, 12]. PA also appears to correlate with attenuated OSA severity and better treatment outcomes [13, 14]. Two previous studies have shown that PA is correlated with reduced daytime sleepiness, as measured by the Epworth Sleepiness Score (ESS) among OSA patients [12, 15]. However, no previous studies have controlled for OSA severity when examining this relationship, although it is possible OSA severity is affected by with physical inactivity and contributes to greater sleep deficits.

This study aimed to describe PA patterns among individuals with OSA and to assess the interaction of PA levels and OSA severity on excessive daytime sleepiness. We hypothesised that PA would be negatively correlated with both OSA severity and excessive daytime sleepiness.

Methods.

## Study design

Data for this cross-sectional study were collected as part of the Sydney Sleep Biobank (SSB) [16]. The SSB includes polysomnography and a comprehensive questionnaire collected from patients of three tertiary hospitals across Sydney (Royal North Shore Hospital, Royal Prince Alfred Hospital and Westmead Hospital) and the Westmead Institute for Medical Research in Australia. Data were collected between August 2018 and June 2022. The SSB recruits patients from tertiary outpatient sleep services referred for polysomnography for any indication. Referrals to these tertiary sleep services are predominantly made from general practice although can also include complex sleep patients referred for a subspecialist opinion by other respiratory and sleep services around the state of New South Wales.

### Participants

**Fig. 1:**
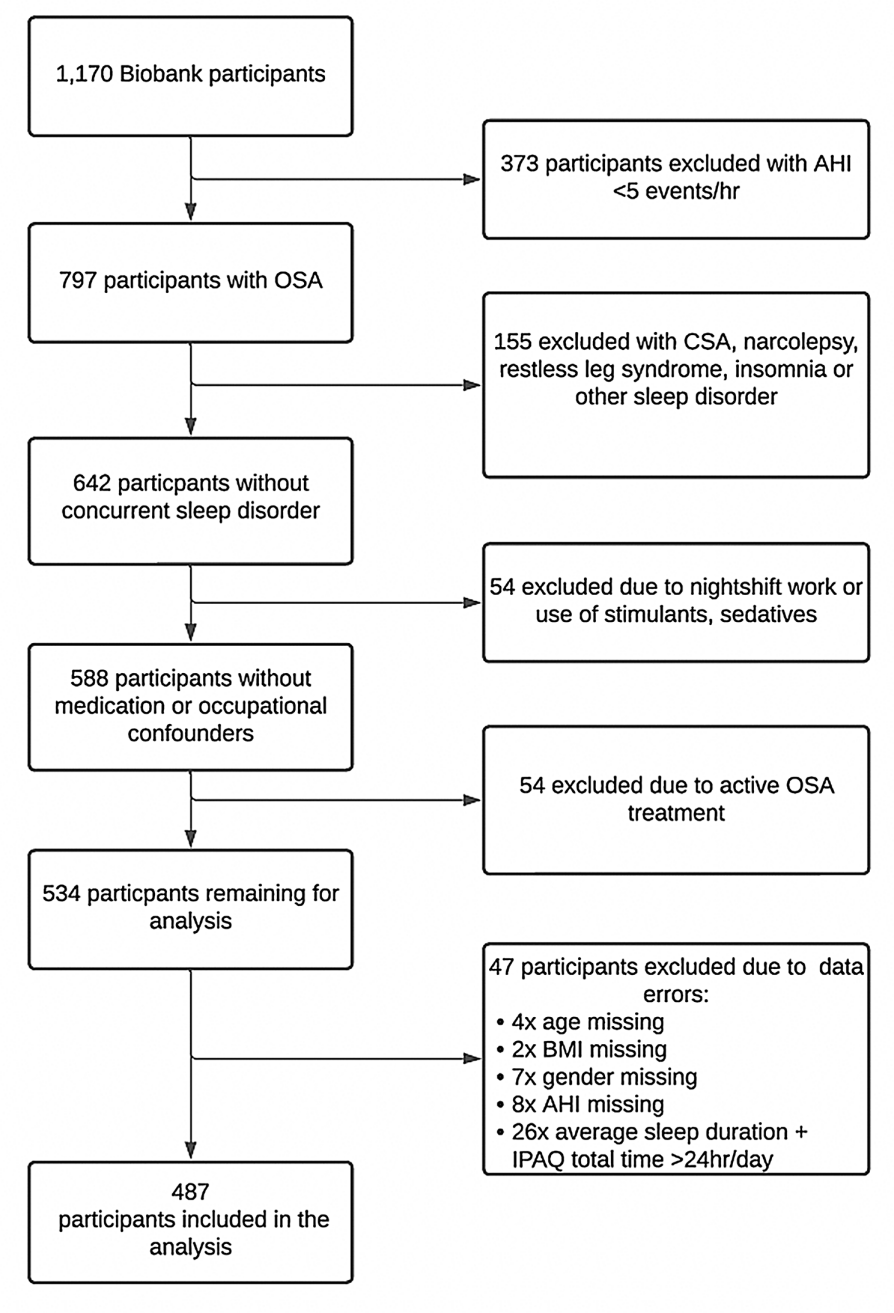
Participant selection flow chart

Participant selection from the Sydney Sleep Biobank for this study is shown in Fig. 1. Participants were included if they were aged 18 years or older with an apnoea-hypopnoea index (AHI) of *≥* 5 events/hour on polysomnography (PSG). We note that for the Biobank as a whole, the most common indication for PSG was suspected obstructive sleep apnoea syndrome (84.2%) with symptoms on referral including daytime fatigue (89.9%), snoring (71.6%), and witnessed apnoeas (42.2%). Participants were excluded if they: (1) reported other sleep disorders which contribute to daytime sleepiness, such as narcolepsy, restless legs syndrome or insomnia; (2) reported an average nightly sleep duration of *≥* 10 or < 4 h; (3) reported being on current OSA therapy; (4) had central events making up more than 20% of their total AHI; (5) reported having any other sleep disturbance including nerve pain, night-time epilepsy, periodic limb movement disorder, sleep paralysis or disturbed sleep from pain; (6) reported use of sedating or stimulating drugs amongst their regular medications, including benzodiazepines, opioids, zolpidem/zopiclone, modafinil/armodafinil or dexamphetamine; (7) were shift workers who performed overnight-shifts (10pm to 8am) as part of their current employment (Fig. 1).

### Measurements

**Fig. 2:**
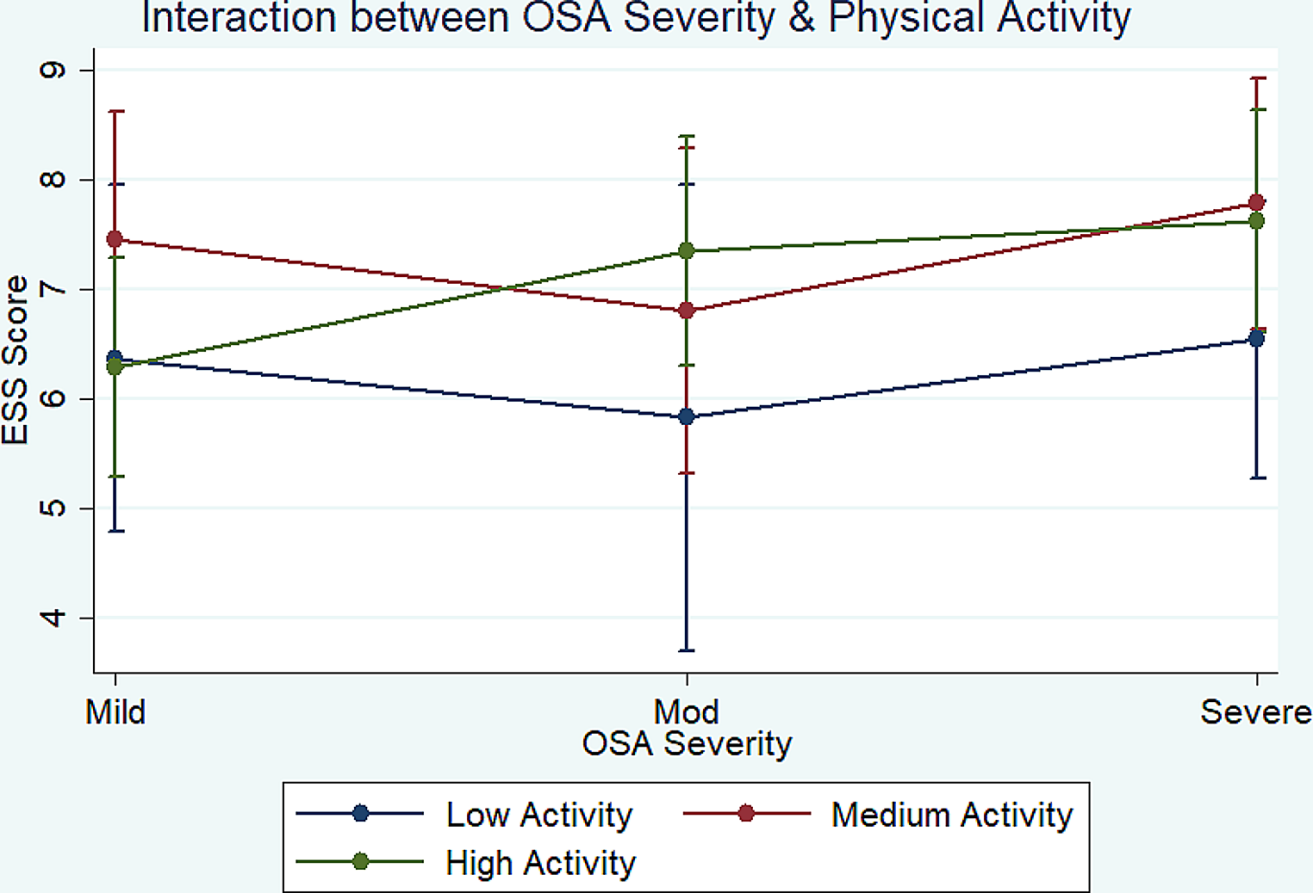
Sleepiness by Obstructive sleep apnoea severity and Level of physical activity. Physical Activity: Low = < 600Met-Min/week; Medium = 600-3,000 Met-Min/Week; High = > 3,000 Met-Min/week. OSA severity: Mild = AHI 5 to < 15 events/h; moderate = 15 to < 30 events/h; severe = ≥ 30 events/h. Abbreviations: OSA, obstructive sleep apnoea. Error bars are 95% confidence intervals

PA was assessed using the International Physical Activity Questionnaire (IPAQ) which measures time spent undertaking varying levels of PA in the preceding seven days and which has been validated for OSA populations [17]. Metabolic equivalent minutes of activity per week were calculated based on responses to this questionnaire as ‘MET-Min/week’ [18, 19]. MET-Min/week were calculated through the addition of all physical activity with one minute of walking equivalent to 3.3 MET-min, one minute of moderate exercise equivalent to 4 MET-Min and one minute vigorous activity equivalent to 8 MET-min [20]. Participants were grouped into categories of ‘Low Physical Activity’ (0-599 MET-Min/week), ‘Moderate Physical Activity’ (600-2,999 MET-Min/week) or ‘High Physical Activity’ (*≥* 3,000 MET-Min/week) [21–23]. 600 MET-Min/week is equivalent to approximately 3 h walking per week, or 2.5 h of moderate activity per week, or 75 min of vigorous activity per week, or a combination of these [22]. The cut-offs were chosen based on the World Health Organisation recommendations for physical activity as well as previously published papers using the IPAQ questionnaire [22, 24]. Physical activity patterns were also examined based on the hours spent sitting, walking, and engaging in moderate to vigorous activity (see Fig. 2).

OSA severity was grouped into three categories of mild (AHI 5-14.9 events/hour), moderate (AHI 15-29.9 events/hour) and severe (AHI *≥* 30 events/hour) based on the American Academy of Sleep Medicine guidelines [25]. Age, sex, body mass index and self-reported sleep duration were considered covariates. Sleep duration was based on question four of the completed Pittsburgh Sleep Quality Index (PSQI) “how many hours of actual sleep do you get at night” which reflects patients’ usual habits during the past month [26].

Excessive daytime sleepiness was assessed using the Epworth Sleepiness Scale (ESS) [27, 28]. ESS is an eight item questionnaire which measures daytime sleepiness with a high degree of unidimensionality [29]. It uses scores ranging from 0 to 24 with scores greater than 10 considered excessively sleepy [28].

### Statistical analysis

All statistical analyses were conducted in Stata 14.2 (StataCorp LP, College Station TX). Differences between the physical activity groups were assessed with one-way ANOVA for continuous variables (age, BMI, sleep duration, AHI, hours of activity and MET-min/week) and chi-squared tests for categorical variables (sex, physical activity group, OSA severity group).

Linear regression analyses were then performed to estimate the effect of physical activity (categorical) and OSA severity (categorical) on ESS (continuous) with adjustment for confounders. Model 0 included OSA severity, sleep duration, age, BMI, and sex as predictors of ESS. Model 1 added physical activity (categorical) to the model. To examine interactions between OSA severity and physical activity on ESS, an interaction term was generated and added to Model 2.

Three sensitivity analyses were conducted as shown in the supplementary materials. The first involved carrying out the regression models including physical activity and OSA severity (AHI) as continuous, rather than categorical, variables. The second sensitivity analysis was performed to assess the removal of BMI from the models to determine whether physical activity influences ESS primarily through obesity, by comparison with the main models presented. We also examined potential sex differences by repeating the Model 1 analysis in males and females separately. Finally, Model 1 was repeated with ESS as a continuous variable.

The significance level was defined as *P* < 0.05 for all analyses.

## Results

Participant characteristics for the sample and by OSA severity group are presented in Table 1. In the sample overall, an average of 41 h of sitting, 7 h of walking, 5 h of moderate-intensity activity, and 4 h of vigorous intensity were reported each week (Table 1). However, there were no differences in mean sitting time, mean walking time, nor mean moderate and vigorous PA time between OSA severity groups.

High levels of overall physical activity were reported by 47% of the sample, moderate levels of physical activity by 32%, and low physical activity by only 21% (Table 1). Overall physical activity differed by OSA severity with more than half of those with mild OSA achieving high levels of physical activity, whilst patients with severe OSA were evenly divided into those with low/medium/high levels of activity (Table 1).

**Table 1:** Participant characteristics by obstructive sleep Apnoea (OSA) severity (*n* = 487)

	Total (*n* = 487)	Mild OSA (*n* = 171)	Moderate OSA (*n* = 125)	Severe OSA (*n* = 191)	Test statistic, *p*-value
Mean AHI (SD)	31.2 (25.3)	9.5 (2.9)	31.4 (4.3)	56.9 (21.7)	F(2,486) = 565.8
					*P* < 0.01
Age in years (SD)	53.7 (14.2)	52.5 (14.7)	54.1 (14.9)	54.3 (14.6)	F(2,486) = 0.81
					*P* = 0.45
Men (n, %)	347 (71.3%)	108 (63.2%)	88 (70.4%)	151 (79.1%)	Chi^2^(2) = 11.19
Women	140 (28.8%)	60 (36.8%)	37 (29.6%)	40 (20.9%)	*P* < 0.01
BMI in kg/m^2^ (SD)	32.5 (8.3)	29.2 (6.3)	31.2 (7.0)	36.2 (9.2)	F (2,486) = 39.90
					*P* < 0.01
Sleep Duration in hours (SD)	6.7 (1.2)	6.9 (1.2)	6.6 (1.0)	6.7 (1.3)	F (2,486) = 3.21
					*P* = 0.04
Physical Activity Level					
-Low (< 600 MET-min/week)	101 (20.7%)	31 (30.7%)	60 (38.7%)	80 (34.6%)	Chi^2^(4) = 14.24
-Medium (600-2,999 MET-min/week)	155 (31.8%)	17 (16.8%)	35 (22.6%)	73 (31.6%)	*P* <0.01
-High (*≥* 3,000 MET-min/week)	231 (47.4%)	53 (52.5%)	60 (38.7%)	78 (33.8%)	
Total Metabolic-Equivalent Minutes/week (SD)	4598.0 (5296.2)	4,517.1 (5,208.6)	4,894.4 (4,306.4)	4,476.4 (5,940.8)	F (2,486) = 0.27
					*P* = 0.77
Sitting Hours/week (SD)	40.8 (25.4)	42.6 (25.2)	38.9 (24.7)	40.4 (26.1)	F (2, 486) = 0.77
					*P* = 0.47
Walking Hours/week (SD)	6.9 (9.2)	7.0 (9.8)	6.7 (6.1)	6.9 (10.2)	F (2,486) = 0.04
					*P* = 0.96
Moderate Activity Hours/week (SD)	4.8 (8.0)	4.63 (8.6)	5.69 (7.8)	4.28 (7.6)	F (2,486) = 1.21
					*P* = 0.30
Vigorous Activity Hours/week (SD)	4.4 (7.6)	4.21 (6.9)	4.60 (5.9)	4.35 (9.1)	F (486) = 0.09
					*P* = 0.91
Moderate-to-Vigorous Activity Hours/week (SD)	9.1 (12.8)	8.8 (12.9)	10.3 (11.0)	8.6 (13.9)	F (2,486) = 0.70
					*P* = 0.50
Mean ESS (SD)	7.0 (4.7)	6.8 (4.5)	7.2 (5.0)	7.1 (4.6)	F (2,486) = 0.43
					*P* = 0.65

Participant characteristics by physical activity group (low/medium/high) are displayed in Table 2.

**Table 2:** Participant characteristics by level of physical activity (*n* = 487)

	Low Physical Activity (*n* = 101)	Medium Physical Activity (*n* = 155)	High Physical Activity (*n* = 231)	Test statistic, *p*-value
Mean AHI (SD) events/h	37.8 (28.1)	30.0 (23.8)	29.0 (24.6)	F (2, 486) = 4.58
				*P* = 0.01
Age in years (SD)	54.3 (14.0)	55.0 (14.3)	52.5 (14.2)	F (2,486) = 1.57
				*P* = 0.21
Men (n, %)	63 (63.4%)	101 (65.2%)	183 (79.2%)	Chi^2^(2) = 13.85
Women	38 (37.6%)	54 (34.8%)	38 (20.8%)	*P* < 0.01
BMI in kg/m^2^ (SD)	35.9 (9.8)	32.2 (8.2)	31.2 (7.2)	F (2, 486) = 12
				*P* < 0.01
Sleep Duration in hours (SD)	6.8 (1.4)	6.8 (1.1)	6.7 (1.2)	F (2, 486) = 0.16
				*P* = 0.8
Mean ESS (SD)	6.2 (5.1)	7.4 (4.5)	7.2 (4.5)	F (2, 486) = 2.39
				*P* = 0.09
Mean Sitting Hours/week (SD)	35.5 (34.0)	45.4 (24.7)	40 (20.6)	F (2, 486) = 5.00
				*P* = 0.01
Mean Walking Hours/week (SD)	0.6 (0.9)	5.5 (3.4)	10.5 (11.8)	F (2, 486) = 52.4
				*P* < 0.01
Mean Moderate Activity Hours/week (SD)	0.0 (0.2)	1.3 (2.2)	9.2 (9.8)	F (2, 486) = 92.1
				*P* < 0.01
Mean Vigorous Activity Hours/week (SD)	0 (0)	0.36 (1.1)	9.0 (9.0)	F (2, 486) = 119.9
				*P* < 0.01
Mean Moderate-to-Vigorous Activity Hours/week (SD)	0.0 (0.2)	1.6 (2.5)	18.1 (13.7)	F (2, 486) = 195.7
				*P* < 0.01

There were no significant differences between physical activity groups on age or sleep duration.

There was a higher proportion of men in the most physically active group compared to the least physically active group. Participants who were the least physically active had significantly higher BMI and AHI than those in the medium and high physical activity groups. Participants who were the least physically active appeared to have lower levels of daytime sleepiness, although this did not reach statistical significance (Table 2).

Table 3 shows the results of the regression modelling. As expected, older age, higher BMI, and shorter sleep duration were independent predictors of ESS scores, while OSA severity was not significantly correlated with ESS (Model 0). When physical activity was added into the regression model, it was found that participants with medium levels of physical activity had significantly higher ESS (compared to those with low levels of physical activity) while no significant difference was observed between those with high and low levels of physical activity (Model 1). Addition of a PA level by OSA severity interaction term did not significantly change the results (Model 2); likelihood ratio test comparing Model 1 (without the interaction term) to Model 2 (with the interaction term) was not statistically significant (LR Chi^2^(4) = 2.75, *P* = 0.60).

**Table 3:** Regression models assessing the association of ESS with physical activity level and OSA severity

ESS difference in unstandardised units							
	Model 0 (95% CI)	*P*	Model 1 (95% CI)^a^	*P*	Model 2 (95% CI)^b^	*P*	
**Gender**							
Male	Reference	-	Reference	-	Reference	-	
Female	0.58 (-0.34,1.50)	0.22	0.60 (-0.33,1.53)	0.2	0.56 (-0.37,1.50)	0.24	
**Age**	-0.04 (-0.07,-0.01)	0.01	-0.04 (-0.07,-0.01)	0.01	-0.03 (-0.06,-0.01)	0.02	
**BMI**	-0.06 (-0.11,-0.01)	0.03	-0.05 (-0.12,0.00)	0.06	-0.05 (-0.10,0.01)	0.09	
**Sleep Duration**	-1.12 (-1.45,-0.78)	< 0.01	-1.12 (-1.43,-0.77)	< 0.01	-1.09 (-1.42,-0.75)	< 0.01	
**OSA Severity**							
Mild	Reference	-	Reference	-	Reference	-	
Moderate	0.29 (-0.75,1.34)	0.59	0.27 (-0.78,1.32)	0.61	-0.54 (-3.19,2.11)	0.69	
Severe	0.74 (-0.29,1.77)	0.16	0.78 (-0.24,1.81)	0.14	0.18 (-1.87,2.22)	0.87	
**PA Level**							
Low			Reference	-	Reference	-	
Medium			1.15 (0.02,2.28)	0.05	1.09 (-0.85,3.03)	0.27	
High			0.79 (-0.30,1.87)	0.15	-0.08 (-1.94,1.78)	0.93	
OSA Severity x PA Level*						0.94	
Moderate OSA x Medium PA					-0.11 (-3.35,3.12)	0.3	
Moderate OSA x High PA					1.59 (-1.41,4.60)	0.91	
Severe OSA x Medium PA					0.15 (-2.41,2.70)	0.25	
Severe OSA x High PA					1.15 (-1.27,3.58)		

*Interaction base levels are Mild OSA and Low Physical activity

Model 0 examines the association between OSA severity and ESS adjusting only for gender, age, BMI and sleep duration

^a^Model 1 additionally adjusts for physical activity level in addition to gender, age, BMI, sleep duration, and OSA severity

^b^Model 2 additionally adjusts for the OSA severity and physical activity interaction term, in addition to gender, age, BMI, sleep duration, OSA severity, and physical activity level

Sensitivity analysis using continuous instead of categorical physical activity and AHI revealed similar results (Supplementary Table 1). Sensitivity analysis without adjustment for BMI showed although the small change in coefficients for physical activity suggests that BMI is partially mediating the relationship between physical activity and sleepiness (Supplementary Table 2).

In the sex-stratified analysis, a clear correlation between increasing physical activity and higher ESS scores was observed exclusively in females, while no such correlation was found in males (Supplementary Table 3a). Additionally, when ESS was analysed as a categorical variable (with ESS *≥* 10 indicating excessive daytime sleepiness and < 10 considered normal), no significant correlation between physical activity and excessive daytime sleepiness was detected (Supplementary Table 4).

## Discussion

This study described physical activity patterns among patients with differing levels of OSA severity, most notably that participants with mild OSA are the most likely to have high levels of physical activity.

Contrary to our hypothesis, we found that physical activity was not significantly associated with excessive daytime sleepiness in the primary multivariate regression model. However, in the sex-stratified model there was a clear correlation in the opposite direction. We had hypothesised that increased physical activity would correlate with decreased ESS scores, based on previous research suggesting that exercise improved sleep quality. Surprisingly, our findings indicated that among women, increased PA was associated with increased ESS scores.

Several factors may explain this unexpected result. Sex differences in sleep architecture and the possibility that women experience an amplified response to circadian modulation induced by physical activity could be contributing factors [30, 31]. Additionally, hormonal differences may also play a role; for instance, testosterone release is more closely correlated with physical activity compared to oestradiol or progesterone [32]. It is conceivable that while physical activity induces a need for sleep and rest in both sexes, this effect in males might be counterbalanced by the post-exercise increase in testosterone [33], particularly given that low testosterone levels in OSA are associated with increased fatigue [34].

Mild OSA may be correlated with higher levels of physical activity for several reasons. Acutely, physical activity has been shown to lead to fluid redistribution, increased muscle tone, and reduced OSA severity [15, 35–39]. Longer-term, physical activity is also linked to weight loss, a key risk factor for OSA severity [15]. It is also likely that reduced physical activity increases obesity and worsens OSA. This is supported by the finding that the least physically active participants had the highest AHI.

The Australian Government’s Department of Health and Aged Care Guidelines on Physical Activity and Exercise recommends that adults (aged 18 to 64) should undertake either 2.5 to 5 h of moderate intensity physical activity per week or 1.25 to 2.5 h of vigorous activity per week or a combination of the two [40]. This would equate to a minimum recommendation of 600 MET-Min/week, meaning that 79% participants in our study reached these recommendations with 82% of mild, 86% of moderate and 72% of severe OSA patients undertaking at least 600 MET-Min/week. Whilst our findings align with previous studies regarding mean moderate-to-vigorous physical activity (MVPA) per week our sample had a much higher mean MET-Min/week and higher proportion of OSA patients in the ‘high physical activity’ group compared to previous literature [22, 41]. It is unclear why this is the case although there may be a healthy volunteer effect but also may reflect the heavy male overrepresentation. Whilst the EPISONO study in Brazil had 56% female population, our cohort was only 28.8% female [22]. Finally, there may be geographic, cultural, and socioeconomic differences in the approaches to exercise in our study compared to previous studies in Sao Paolo, Brazil [22] or Al-Hassa, Saudi Arabia [41].

These findings also suggest that ESS is not a reliable indicator of OSA severity and perhaps other OSA metrics such as arousal intensity or hypoxic burden may better predict OSA severity [42]. ESS measures “tendency to fall asleep” which is distinct from the drowsiness/sleepiness without unexpected somnolence caused by OSA. OSA is also phenotypically heterogenous and disease severity may only be linked to sleepiness in the ‘Excessively Sleepy’ phenotype [43]. The low mean ESS (mean ESS 7.0, SD 4.7) may also have caused a ceiling effect contributing to a lack of correlation between ESS with physical activity and OSA severity. Finally, it is possible that unmeasured confounding factors such caffeine, alcohol intake, and diet are affecting the relationship [44–46].

It was also curious that participants with the lowest level of physical activity had significantly lower ESS than those in the medium physical activity group. Previous research suggests exercise is associated with shorter sleep latency and longer sleep duration so it had been presumed that this would lead to reduced ESS [12, 15, 47]. It may be, however, that physical activity necessitates longer sleep for recovery in order to achieve the same level of wakefulness [48, 49]. Finally, it is possible that high levels of physical activity induce mild hypoglycaemia, and the resultant fatigue contributed to participants’ reporting of sleepiness [50].

The strengths of this study are that it controlled for multiple confounders including medications, comorbid sleep disorders, and shift work, which has not been done in previous studies on this question. It also had a large sample size of 487 participants recruited from more than one site [15].

The main limitation of this study is the cross-sectional study design which prevents inference of a causal relationship between physical activity, OSA severity, and sleepiness. It is likely that there is a bidirectional relationship between physical activity and sleepiness in which physical activity may induce or reduce sleepiness but sleepiness may also limit a person’s ability to undertake physical activity. The study is also limited by reliance on self-reported physical activity which is prone to bias; previous studies suggest that patients may overestimate their physical activity [51]. Finally, the time of day that physical activity occurred was unable to be recorded and it may be that evening physical activity was detrimental to sleep quality whilst morning activity was beneficial to wakefulness [52, 53]. Future research should also employ objective measures of physical activity whilst utilising a longitudinal study design to elucidate the direction of causality between activity and sleepiness.

In conclusion, this study brings to light the physical activity patterns in patients with differing levels of OSA severity, showing no mean difference in time spent sitting, walking, or undertaking moderate-to-vigorous physical activity across OSA severity groups, but that more patients with mild OSA engaged in high levels of physical activity as measured by metabolic-equivalent minutes of activity. Whilst physical activity is important for a myriad of health benefits it does not appear to be associated with reduced sleepiness symptoms amongst OSA patients; sleepiness was primarily accounted for by differences in age, obesity, and sleep duration across OSA severity groups. There may be sex differences as physical activity was associated with reduced sleepiness in women only and this unexpected finding will need to be replicated in future studies. More research is needed with objective measures of physical activity and varied metrics of assessing the clinical impact of OSA beyond ESS scores.

## Electronic supplementary material

Below is the link to the electronic supplementary material.

Supplementary Material 1

## Data Availability

Access to the datasets for this study may be available through application to the Sydney Sleep Biobank. Such requests should be directed to the corresponding author.

## Abbreviations

AHI: Apnoea-Hypopnoea Index
ANOVA: Analysis of Variance
BMI: Body Mass Index
ESS: Epworth Sleepiness Scale
HREC: Human Research Ethics Committee
IPAQ: International Physical Activity Questionnaire
LR: Likelihood Ratio
MET-Min/week: Metabolic Equivalent Minutes of Activity per Week
MPVA: Moderate to Vigorous Physical Activity
NSLHD: Northern Sydney Local Health District
OSA: Obstructive Sleep Apnoea
PSG: Polysomnography
PSQI: Pittsburgh Sleep Quality Index
REM: Rapid Eye movement
SSA: Site Specific Approval
SSB: Sydney Sleep Biobank
TX: Texas
WHO: World Health Organisation

## Declarations

### Institution where work was performed

Data for this study were collected as part of the Sydney Sleep Biobank (SSB). The SSB includes polysomnography and a comprehensive questionnaire collected from patients of three tertiary hospitals across Sydney (Royal North Shore Hospital, Royal Prince Alfred Hospital and Westmead Hospital) and the Westmead Institute for Medical Research in Australia.

## References

[1] Patil SP, Ayappa IA, Caples SM, Kimoff RJ, Patel SR, Harrod CG (2019) Treatment of adult obstructive sleep apnea with positive airway pressure: an American academy of sleep medicine clinical practice guideline. J Clin Sleep Med 15(2):335–343 30736887 10.5664/jcsm.7640 PMC6374094

[2] Benjafield AV, Ayas NT, Eastwood PR, Heinzer R, Ip MS, Morrell MJ et al (2019) Estimation of the global prevalence and burden of obstructive sleep apnoea: a literature-based analysis. Lancet Respiratory Med 7(8):687–698 10.1016/S2213-2600(19)30198-5 PMC7007763 31300334

[3] Sateia MJ (2014) International classification of sleep disorders. Chest 146(5):1387–1394 25367475 10.1378/chest.14-0970

[4] Kubitz KA, Landers DM, Petruzzello SJ, Han M (1996) The effects of acute and chronic exercise on sleep: a meta-analytic review. Sports Med 21:277–291 8726346 10.2165/00007256-199621040-00004

[5] Youngstedt SD, O’Connor PJ, Dishman RK (1997) The effects of acute exercise on sleep: a quantitative synthesis. Sleep 20(3):203–214 9178916 10.1093/sleep/20.3.203

[6] Uchida S, Shioda K, Morita Y, Kubota C, Ganeko M, Takeda N (2012) Exercise effects on sleep physiology. Front Neurol 3:48 22485106 10.3389/fneur.2012.00048 PMC3317043

[7] Vincent GE, Sargent C, Roach GD, Miller DJ, Kovac K, Scanlan AT et al (2020) Exercise before bed does not impact sleep inertia in young healthy males. J Sleep Res 29(3):e12903 31621995 10.1111/jsr.12903

[8] Chennaoui M, Arnal PJ, Sauvet F, Léger D (2015) Sleep and exercise: a reciprocal issue? Sleep Med Rev 20:59–72 25127157 10.1016/j.smrv.2014.06.008

[9] Dunn AL, Trivedi MH, Kampert JB, Clark CG, Chambliss HO (2005) Exercise treatment for depression: efficacy and dose response. American journal of preventive medicine;28(1):1–8 10.1016/j.amepre.2004.09.003 15626549

[10] Oh C-M, Kim HY, Na HK, Cho KH, Chu MK (2019) The effect of anxiety and depression on sleep quality of individuals with high risk for insomnia: a population-based study. Front Neurol 10:849 31456736 10.3389/fneur.2019.00849 PMC6700255

[11] Osailan AM, Elnaggar RK, Alsubaie SF, Alqahtani BA, Abdelbasset WK (2021) The association between cardiorespiratory fitness and reported physical activity with sleep quality in apparently healthy adults: a cross-sectional study. Int J Environ Res Public Health 18(8):4263 33920540 10.3390/ijerph18084263 PMC8072608

[12] Çalışkan H, Ertürk N, Kütükçü EÇ, Arıkan H, Yağlı NV, Sağlam M et al (2019) The relationship between the physical activity level and fatigue perception, quality of life and psychological status in patients with obstructive sleep apnea syndrome. J Turkish Sleep Med 6(1):1

[13] Qamar A, Qureshi MA, Nazar S, Baig S, Iffat W, Owais M (2019) Physical activity and Apnoea-hypopnea index in obstructive sleep Apnoea. Pakistan J Physiol 15(3):67–70

[14] Jean RE, Duttuluri M, Gibson CD, Mir S, Fuhrmann K, Eden E et al (2017) Improvement in physical activity in persons with obstructive sleep apnea treated with continuous positive airway pressure. J Phys Activity Health 14(3):176–182 10.1123/jpah.2016-0289 27997271

[15] Silva RPd, Martinez D, Bueno KSS, Uribe-Ramos JM (2019) Effects of exercise on sleep symptoms in patients with severe obstructive sleep apnea. Jornal Brasileiro de Pneumologia 45 10.1590/1806-3713/e20180085 PMC6715025 31241653

[16] Cistulli P (2023) Sydney Sleep Biobank: University of Sydney: Charles Perkins Centre; [Available from: https://www.sydney.edu.au/charles-perkins-centre/our-research/sleep/sydney-sleep-biobank.html

[17] Adolphs MW The Validity and Reliability of the PAVS and IPAQ-SF as Physical Activity Assessment Tools in Patients with Obstructive Sleep Apnea 2020

[18] Booth ML, Ainsworth BE, Pratt M, Ekelund U, Yngve A, Sallis JF et al (2003) International physical activity questionnaire: 12-country reliability and validity. Med Sci Sports Exerc 195(9131/03):3508–1381 10.1249/01.MSS.0000078924.61453.FB 12900694

[19] Guidelines for data processing and analysis of the International Physical Activity Questionnaire (IPAQ)-short and long forms: IPAQ Research Committee (2005) [Available from: http://www.ipaq.ki.se/scoring.pdf

[20] Craig CL, Marshall AL, Sjöström M, Bauman AE, Booth ML, Ainsworth BE et al (2003) International physical activity questionnaire: 12-country reliability and validity. Medicine & Science in Sports & Exercise 35(8):1381-95 10.1249/01.MSS.0000078924.61453.FB 12900694

[21] Bull FC, Al-Ansari SS, Biddle S, Borodulin K, Buman MP, Cardon G et al (2020) World health organization 2020 guidelines on physical activity and sedentary behaviour. Br J Sports Med 54(24):1451–1462 10.1136/bjsports-2020-102955 PMC7719906 33239350

[22] Mônico-Neto M, Antunes HKM, Dos Santos RVT, D’Almeida V, de Souza AAL, Bittencourt LRA et al (2018) Physical activity as a moderator for obstructive sleep Apnoea and cardiometabolic risk in the EPISONO study. Eur Respir J 52(4) 10.1183/13993003.01972-2017 30093572

[23] Zota IM, Roca M, Leon MM, Cozma CD, Anghel L, Statescu C et al (2023) Long-Term Adherence in Overweight Patients with Obstructive Sleep Apnea and Hypertension—A Pilot Prospective Cohort Study. Diagnostics;13(8):1447 10.3390/diagnostics13081447 PMC10137954 37189548

[24] World Health Organization t (2010) Global recommendations on physical activity for health. World Health Organization 26180873

[25] Kapur VK, Auckley DH, Chowdhuri S, Kuhlmann DC, Mehra R, Ramar K et al (2017) Clinical practice guideline for diagnostic testing for adult obstructive sleep apnea: an American academy of sleep medicine clinical practice guideline. J Clin Sleep Med 13(3):479–504 28162150 10.5664/jcsm.6506 PMC5337595

[26] Buysse DJ, Reynolds CF III, Monk TH, Berman SR, Kupfer DJ (1989) The Pittsburgh sleep quality index: a new instrument for psychiatric practice and research. Psychiatry Res 28(2):193–213 2748771 10.1016/0165-1781(89)90047-4

[27] Johns MW (1992) Reliability and factor analysis of the Epworth sleepiness scale. Sleep 15(4):376–381 1519015 10.1093/sleep/15.4.376

[28] Johns MW (1991) A new method for measuring daytime sleepiness: the Epworth sleepiness scale. Sleep 14(6):540–545 1798888 10.1093/sleep/14.6.540

[29] Lapin BR, Bena JF, Walia HK, Moul DE (2018) The Epworth sleepiness scale: validation of one-dimensional factor structure in a large clinical sample. J Clin Sleep Med 14(8):1293–1301 30092893 10.5664/jcsm.7258 PMC6086944

[30] Kumar S, Anton A, D’Ambrosio CM (2021) Sex differences in obstructive sleep apnea. Clin Chest Med 42(3):417–425 34353448 10.1016/j.ccm.2021.04.004

[31] Santhi N, Lazar AS, McCabe PJ, Lo JC, Groeger JA, Dijk DJ (2016) Sex differences in the circadian regulation of sleep and waking cognition in humans. Proc Natl Acad Sci U S A 113(19):E2730–E2739 27091961 10.1073/pnas.1521637113 PMC4868418

[32] Shahid W, Noor R, Bashir MS (2024) Effects of exercise on sex steroid hormones (estrogen, progesterone, testosterone) in eumenorrheic females: A systematic to review and meta-analysis. BMC Womens Health 24(1):354 38890710 10.1186/s12905-024-03203-y PMC11186217

[33] Ahmadi MA, Zar A, Krustrup P, Ahmadi F (2018) Testosterone and cortisol response to acute intermittent and continuous aerobic exercise in sedentary men. Sport Sci Health 14:53–60

[34] Bercea RM, Mihaescu T, Cojocaru C, Bjorvatn B (2015) Fatigue and serum testosterone in obstructive sleep apnea patients. The clinical respiratory journal 9(3):342-9 10.1111/crj.12150 24725752

[35] Redolfi S, Bettinzoli M, Venturoli N, Ravanelli M, Pedroni L, Taranto-Montemurro L et al (2015) Attenuation of obstructive sleep apnea and overnight rostral fluid shift by physical activity. Am J Respir Crit Care Med 191(7):856–858 25830523 10.1164/rccm.201412-2192LE

[36] Mendelson M, Lyons OD, Yadollahi A, Inami T, Oh P, Bradley TD (2016) Effects of exercise training on sleep Apnoea in patients with coronary artery disease: a randomised trial. Eur Respir J 48(1):142–150 27076578 10.1183/13993003.01897-2015

[37] Frange C, Elias RM, Siengsukon C, Coelho FMS (2023) Physical activity for obstructive sleep apnea after stroke? A pilot study assessing the contribution of body fluids. Sleep Breath 27(4):1343–1350 36327028 10.1007/s11325-022-02735-7

[38] Iftikhar IH, Kline CE, Youngstedt SD (2014) Effects of exercise training on sleep apnea: a meta-analysis. Lung 192:175–184 24077936 10.1007/s00408-013-9511-3 PMC4216726

[39] Kline CE, Crowley EP, Ewing GB, Burch JB, Blair SN, Durstine JL et al (2011) The effect of exercise training on obstructive sleep apnea and sleep quality: a randomized controlled trial. Sleep 34(12):1631–1640 22131599 10.5665/sleep.1422 PMC3208839

[40] Physical activity and exercise guidelines for all Australians: Department of Health and Aged Care (2021) [updated 10/05/2021. Available from: https://www.health.gov.au/topics/physical-activity-and-exercise/physical-activity-and-exercise-guidelines-for-all-australians/for-adults-18-to-64-years

[41] Amin TT, Suleman W, Ali A, Gamal A, Al Wehedy A (2011) Pattern, prevalence, and perceived personal barriers toward physical activity among adult Saudis in Al-Hassa, KSA. J Phys Activity Health 8(6):775–784 10.1123/jpah.8.6.775 21832292

[42] Malhotra A, Ayappa I, Ayas N, Collop N, Kirsch D, Mcardle N et al (2021) Metrics of sleep apnea severity: beyond the apnea-hypopnea index. Sleep 44(7):zsab030 33693939 10.1093/sleep/zsab030 PMC8271129

[43] Keenan BT, Kim J, Singh B, Bittencourt L, Chen N-H, Cistulli PA et al (2018) Recognizable clinical subtypes of obstructive sleep apnea across international sleep centers: a cluster analysis. Sleep 41(3):zsx214 29315434 10.1093/sleep/zsx214 PMC5914381

[44] Wheaton AG, Perry GS, Chapman DP, Croft JB (2012) Sleep disordered breathing and depression among US adults: National Health and Nutrition Examination Survey, 2005–2008. Sleep 35(4):461-7 10.5665/sleep.1724 PMC3296787 22467983

[45] Chen Y-H, Keller JK, Kang J-H, Hsieh H-J, Lin H-C (2013) Obstructive sleep apnea and the subsequent risk of depressive disorder: a population-based follow-up study. Journal of Clinical Sleep Medicine 9(5):417– 23 10.5664/jcsm.2652 PMC3629313 23674930

[46] Grandner MA, Min JS, Saad R, Leary EB, Eldemir L, Hyman D (2023) Health-related impact of illness associated with excessive daytime sleepiness in patients with obstructive sleep apnea. Postgrad Med 135(5):501–510 37129416 10.1080/00325481.2023.2203623

[47] Kline CE, Ewing GB, Burch JB, Blair SN, Durstine JL, Davis JM et al (2012) Exercise training improves selected aspects of daytime functioning in adults with obstructive sleep apnea. J Clin Sleep Med 8(4):357–365 22893765 10.5664/jcsm.2022 PMC3407253

[48] King AC, Oman RF, Brassington GS, Bliwise DL, Haskell WL (1997) Moderate-intensity exercise and self-rated quality of sleep in older adults: a randomized controlled trial. JAMA 277(1):32–37 8980207

[49] Huang B-H, Hamer M, Duncan MJ, Cistulli PA, Stamatakis E (2021) The bidirectional association between sleep and physical activity: A 6.9 years longitudinal analysis of 38,601 UK biobank participants. Prev Med 143:106315 33171179 10.1016/j.ypmed.2020.106315

[50] Nybo L, Møller K, Pedersen B, Nielsen B, Secher N (2003) Association between fatigue and failure to preserve cerebral energy turnover during prolonged exercise. Acta Physiol Scand 179(1):67–74 12940940 10.1046/j.1365-201X.2003.01175.x

[51] Ahmad MH, Salleh R, Mohamad Nor NS, Baharuddin A, Rodzlan Hasani WS, Omar A et al (2018) Comparison between self-reported physical activity (IPAQ-SF) and pedometer among overweight and obese women in the MyBFF@ home study. BMC Womens Health 18:85–90 30066635 10.1186/s12905-018-0599-8 PMC6069802

[52] Stutz J, Eiholzer R, Spengler CM (2019) Effects of evening exercise on sleep in healthy participants: a systematic review and meta-analysis. Sports Med 49(2):269–287 30374942 10.1007/s40279-018-1015-0

[53] Frimpong E, Mograss M, Zvionow T, Dang-Vu TT (2021) The effects of evening high-intensity exercise on sleep in healthy adults: A systematic review and meta-analysis. Sleep Med Rev 60:101535 34416428 10.1016/j.smrv.2021.101535

