# Supplementary material for: Patterns of physical activity in Obstructive Sleep Apnoea and their association with sleepiness
Source: Sleep Breath. 2025 Apr 4;29(2):147. doi: 10.1007/s11325-025-03314-2 (PMC11971212; doi:10.1007/s11325-025-03314-2)
Supplement: Supplementary file 1 — Supplementary Material 1 [file 11325_2025_3314_MOESM1_ESM.docx]

Supplementary Materials

**Supplementary Table 1. Regression Models Using Continuous Physical Activity (increments of 100 MET-min/week) and OSA Severity (increments of 10 AHI)**

| ESS Difference in unstandardised units | | | | | | |
| --- | --- | --- | --- | --- | --- | --- |
|  | Model 0 (95% CI) | *P* | Model 1 (95% CI)^a^ | *P* | Model 2 (95% CI)^b^ | *P* |
| BMI | -0.07 (-0.12,-0.01) | 0.03 | -0.07 (-0.13,-0.01) | 0.02 | -0.07 (-0.13,-0.01) | 0.09 |
| Gender  Male  Female | Reference  0.58 (-0.34,1.50) | -  0.21 | Reference  0.54 (-0.40,1.47) | -  0.26 | Reference  0.50 (-0.44,1.44) | -  0.30 |
| Age | -0.04 (-0.07,-0.01) | 0.01 | -0.04 (-0.07,-0.01) | 0.01 | -0.04 (-0.07,-0.01) | 0.02 |
| Sleep Duration | -1.12 (-1.45,-0.79) | <0.01 | -1.13 (-1.46,-0.80) | <0.01 | -1.14 (-1.47,-0.80) | <0.01 |
| AHI (x10) | 0.14 (-0.05,0.33) | 0.15 | 0.14 (-0.05,0.32) | 0.15 | 0.08 (-0.15,0.31) | 0.51 |
| MET-min/week (x100) |  |  | -0.02 (-0.10,0.06) | 0.59 | -0.07 (-0.19,0.06) | 0.31 |
| AHI (x10) x MET-min/week (x100) |  |  |  |  | 0.01 (-0.02,0.04) | 0.39 |

Model analysed with and without the interaction term (Model 1 vs. Model 2) revealed an LR chi^2^(1) = 0.76 and P = 0.38.

Model 0 examines the association between OSA severity (measured in increments of 10 AHI) and ESS adjusting only for gender, age, BMI and sleep duration.

^a^Model 1 additionally adjusts for physical activity level (measured in increment of 100 Met-min/week) in addition to gender, age, BMI, sleep duration, and OSA severity

^b^Model 2 additionally adjusts for the OSA severity and physical activity interaction term, in addition to gender, age, BMI, sleep duration, OSA severity, and physical activity level

**Supplementary Table 2. Sensitivity Analysis with and without adjustment for BMI**

Sensitivity analysis was done to compare models with and without BMI to assess whether BMI mediated the relationship between physical activity and sleepiness.

| ESS Difference in unstandardised units | | | | |
| --- | --- | --- | --- | --- |
|  | Model 1 without BMI (95% CI) | *P* | Model 1 with BMI (95% CI) | *P* |
| Gender  Male  Female | Reference  0.42 (-0.49,1.32) | -  0.37 | Reference  0.60 (-0.33,1.53) | -  0.20 |
| Age | -0.03 (-0.6,0.00) | 0.03 | -0.04 (-0.07,-0.01) | 0.01 |
| Sleep Duration | -1.06 (-1.39,-0.73) | <0.01 | -1.11 (-1.44,-0.77) | <0.01 |
| OSA Severity  Mild  Moderate  Severe | Reference  0.15 (-0.90,1.19)  0.39 (-0.55,1.34) | -  0.78  0.41 | Reference  0.27 (-0.78,1.32)  0.78 (-0.24,1.81) | -  0.61  0.14 |
| PA Level  Low  Medium  High | Reference  1.29 (0.16,2.42)  0.97 (-0.11,2.04) | -  0.03  0.08 | Reference  1.15 (0.02,2.28)  0.79 (-0.30,1.88) | -  0.05  0.15 |
| BMI |  |  | -0.05 (-0.11,0.00) | 0.06 |

Comparison of models with and without BMI revealed an LR chi^2^(1) = 3.56 and P = 0.06.

Model 1 is a regression models assessing the association of ESS and OSA severity whilst adjusting for physical activity level in addition to gender, age, BMI, sleep duration

Supplementary Table 3a. Regression Model 1 Analysis Stratified by Sex

The relationship of OSA severity and PA with sleepiness was examined separately in both males and in females.

| ESS Difference in unstandardised units | | | | |
| --- | --- | --- | --- | --- |
|  | Model 1 males only (95% CI) | *P* | Model 1 females only (95% CI) | *P* |
| Age | -0.09 (-0.15, -0.03) | 0.01 | -0.02 (-0.05,0.02) | 0.33 |
| BMI | -0.02 (-0.12,0.07) | 0.61 | -0.06 (-0.13, 0.01) | 0.09 |
| Sleep Duration | -1.11 (-1.71,-0.52) | <0.01 | -1.20 (-1.61, -0.78) | <0.01 |
| OSA Severity  Mild  Moderate  Severe | Reference  -1.10 (-3.03,0.84)  -0.19 (-2.24,1.86) | 0.26  0.85 | Reference  0.54 (-0.42, 2.09)  1.23 (0.04,2.43) | 0.19  0.04 |
| PA Level  Low  Medium  High | Reference  0.46 (-1.52, 2.44)  -0.09 (-2.11,1.92) | 0.65  0.93 | Reference  1.54 (0.14, 2.95)  1.32 (0.02, 2.63) | 0.03  0.05 |

Supplementary Table 3b. Regression Model 2Analysis Stratified by Sex

The relationship of OSA severity and PA with sleepiness was examined separately in both males and in females including an interaction term for OSA severity and physical activity.

| ESS Difference in unstandardised units | | | | |
| --- | --- | --- | --- | --- |
|  | Model 2 males only (95% CI) | *P* | Model 2 females only (95% CI) | *P* |
| Age | -0.09 (-0.15, -0.02) | <0.01 | -0.01 (-0.05, 0.02) | 0.39 |
| BMI | -0.02 (-0.12, 0.07) | 0.62 | -0.06 (-0.13, 0.01) | 0.11 |
| Sleep Duration | -1.10 (-1.71, -0.50) | <0.01 | -1.17 (-1.59, -0.08) | <0.01 |
| OSA Severity  Mild  Moderate  Severe | Reference  -1.48 (-5.74, 2.81)  -0.20 (-3.65, 3.25) | 0.50  0.91 | Reference  -0.05 (-3.47, 3.37)  0.75 (-1.82, 3.32) | 0.98  0.56 |
| PA Level  Low  Medium  High | Reference  0.48 (-2.53, 3.49)  -0.36 (-3.57, 2.85) | 0.75  0.83 | Reference  1.66 (-0.92, 4.24)  0.57 (-1.77, 2.90) | 0.21  0.63 |
| OSA Severity x PA Level*  Moderate OSA x Medium PA  Moderate OSA x High PA  Severe OSA x Medium PA  Severe OSA x High PA | 1.32 (-3.79, 6.42)  -0.20 (-5.41, 5.01)  -1.78 (-6.42, 2.86)  1.59 (-3.06, 6.23) | 0.61  0.94  0.45  0.50 | -0.88 (-5.09, 3.32)  1.81 (-1.96, 5.59)  0.17 (-3.04, 3.38)  0.70 (-2.24, 4.65) | 0.68  0.35  0.92  0.64 |

*Interaction base levels are Mild OSA and Low Physical activity

Supplementary Table 4. Regression Analysis using ESS as a continuous and binary variable

This analysis compares using excessive daytime sleepiness (EDS) as a categorical vs continuous variable with EDS defined as ESS >10 (compared to ESS <10)

|  | Model 1 linear regression with continuous EDS (95% CI) | *P* | Model 1 logistic regression with binary EDS (95% CI) | *P* |
| --- | --- | --- | --- | --- |
|  | ESS Difference in unstandardised units | | Odds of EDS | |
| Gender  Male  Female | Reference  0.60 (-0.33,1.53) | -  0.20 | Reference  1.17 (0.73, 1.89) | 0.51 |
| Age | -0.04 (-0.07,-0.01) | 0.01 | 0.98 (0.97, 1.00) | 0.03 |
| BMI | -0.05 (-0.12,0.00) | 0.06 | 0.99 (0.96, 1.01) | 0.34 |
| Sleep Duration | -1.12 (-1.43,-0.77) | <0.01 | 0.63 (0.53, 0.76) | <0.01 |
| OSA Severity  Mild  Moderate  Severe | Reference  0.27 (-0.78,1.32)  0.78 (-0.24,1.81) | -  0.61  0.14 | Reference  1.19 (0.70, 2.04)  1.16 (0.68, 1.98) | 0.51  0.60 |
| PA Level  Low  Medium  High | Reference  1.15 (0.02,2.28)  0.79 (-0.30,1.87) | -  0.05  0.15 | Reference  1.15 (0.64, 2.09)  1.05 (0.59, 1.87) | 0.64  0.86 |
